# Efficacy and Safety of Single- Versus Double-Unit Red Blood Cell Transfusion in Trauma Patients With Asymptomatic Anemia

**DOI:** 10.7759/cureus.114534

**Published:** 2026-08-14

**Authors:** Greggory R Davis, Shahrzad Talebinejad, Rutger Fury, Christi Kruger, Richard H Lewis

**Affiliations:** 1 Emergency Medicine, Louisiana State University Health Sciences Center, Baton Rouge, USA; 2 Trauma Surgery, Our Lady of the Lake Regional Medical Center, Baton Rouge, USA; 3 Medicine, Louisiana State University Health Sciences Center New Orleans, New Orleans, USA

**Keywords:** asymptomatic anemia, packed red blood cells (prbc), packed red blood cell transfusion, retrospective research, transfusion in trauma, trauma

## Abstract

Introduction

Optimal packed red blood cell (PRBC) transfusion strategies for stable, non-bleeding anemic trauma patients remain controversial. The goal of this study was to examine the need for repeat transfusion following a single-unit (one unit) or double-unit (two units) transfusion strategy in trauma patients with asymptomatic anemia.

Methods

A single-site retrospective study at a level 1 trauma center was conducted in trauma patients with asymptomatic anemia requiring transfusion during index admission. Patient characteristics, admission vital signs, lab values, total PRBC transfusions, and operative interventions were recorded. Outcomes, including the need for additional transfusions beyond the initial anemia transfusion, changes in hemoglobin/hematocrit levels, hospital length of stay (LOS), ICU LOS, and total number of PRBC units transfused, were collected. Multivariable logistic regression was then performed to determine the impact of transfusion strategy on the need for subsequent transfusion.

Results

A total of 344 patients were identified: 114 received a double-unit PRBC, and 230 received a single-unit PRBC for asymptomatic anemia. The double-unit transfusion group received more blood transfusions than the single-unit group (4 vs. 3 units, p = 0.005). However, the need for additional transfusions did not differ between groups (42% vs. 39%, p = 0.6). Multivariable logistic regression identified only post-transfusion operative intervention as an independent predictor of repeat transfusion with an adjusted odds ratio (95% CI) of 6.55 (3.99, 10.76; p < 0.001). After adjusting for post-transfusion operation, most recent hemoglobin, and admission Glasgow Coma Scale score, the initial transfusion strategy did not significantly affect the need for repeat transfusion, with an adjusted odds ratio (95% CI) of 0.93 (0.54, 1.59; p = 0.786).

Conclusions

Restrictive, single-unit transfusion strategies appear safe and effective in stable, non-bleeding trauma patients. A single-unit transfusion strategy did not increase the likelihood for the need for repeat transfusions. These findings suggest that higher initial transfusion doses may unnecessarily increase blood product exposure without improving clinical outcomes.

## Introduction

Packed red blood cell (PRBC) transfusion remains a vital component of trauma care, particularly in the management of post-injury anemia. Trauma patients are uniquely susceptible to anemia due to hemorrhage, hemodilution, repeated phlebotomy, and the inflammatory response to injury [1]. Anemia in this setting can compromise oxygen delivery, delay tissue healing, and increase the risk of morbidity and mortality. However, while transfusion can correct low hemoglobin (Hgb), evidence suggests that more transfusion does not necessarily equate to better outcomes. Furthermore, in critically ill populations, anemia is nearly ubiquitous but not inherently dangerous. It is not the Hgb level alone that should guide transfusion decisions, but rather the patient’s overall clinical picture. As emphasized in early foundational work, transfusions should not be administered solely to correct laboratory values in the absence of symptoms or clear physiologic compromise [1]. This principle aligns with growing evidence across various patient populations, including those with active bleeding.

Previous guidelines from the Association for the Advancement of Blood & Biotherapies (AABB; formerly the American Association of Blood Banks) endorsed liberal transfusion thresholds, for example, initiating transfusion at an Hgb level below 10 g/dL [2]. However, numerous randomized controlled trials (RCTs) and systematic reviews have since challenged this convention, finding no survival benefit from liberal transfusion and suggesting increased morbidity and resource utilization [3,4]. Thus, contemporary guidelines now favor restrictive transfusion thresholds (typically 7 g/dL) and advocate for single-unit transfusions with interval reassessment, particularly in stable, non-bleeding patients [5,6].

Despite growing consensus favoring restrictive transfusion thresholds, uncertainty remains regarding the optimal transfusion strategy in certain clinical contexts. For example, a recent RCT found that a liberal transfusion strategy (Hgb < 9 g/dl) was superior to a restrictive strategy (Hgb < 7 g/dl) in the context of traumatic brain injury (TBI) [7]. In addition, decisions to transfuse at higher Hgb thresholds or to administer multiple units, rather than a single unit, reassess approach, are often influenced by provider preference, institutional culture, and clinical presentation rather than definitive evidence. In particular, the appropriate transfusion dose and threshold remain poorly defined in patients who are not actively bleeding or overtly unstable but still require resuscitation or perioperative support. This uncertainty is particularly important in the trauma population, which often includes younger, hemodynamically stable patients who may tolerate anemia better than other critically ill cohorts. Moreover, trauma patients often undergo multiple surgical interventions and are subject to complex resuscitation dynamics that can influence transfusion decisions. Given the potential harms of unnecessary transfusion, including alloimmunization, transfusion-associated circulatory overload (TACO), and transfusion-related acute lung injury (TRALI), a clearer understanding of transfusion dose efficacy in trauma is critical [8]. Thus, this retrospective cohort study sought to compare the need for repeat transfusions in hemodynamically stable trauma patients without acute bleeding who received single- or double-unit PRBC transfusions as an initial treatment strategy for asymptomatic anemia. Secondarily, we sought to compare mortality, hospital length of stay (LOS), ICU LOS, total PRBC units transfused, wound infections, and transfusion reaction differences between groups.

## Materials and methods

In this retrospective observational cohort study, consecutive patients who underwent transfusion for asymptomatic anemia from January 1, 2022, to February 28, 2024, were identified from the trauma registry following approval from the institutional review board. The charts of these patients were reviewed for data regarding patient characteristics, mechanism and severity of injury, severity of shock, and outcomes, including need for additional transfusion and total number of PRBC units transfused. To evaluate the impact of transfusion volume on outcomes, those who received one unit of PRBC for asymptomatic anemia were compared to those who received two units. Need for additional transfusion beyond the index single- or double-unit transfusion was the primary outcome. Total number of PRBC units transfused during admission, operative interventions, hospital LOS, ICU LOS, wound infections, transfusion reactions, and mortality were secondary outcomes.

Patients admitted between January 1, 2022, and February 28, 2024, with hemoglobin levels between < 7 g/dL and ≥ 6 g/dL prior to receiving either single- or double-unit blood transfusions were included. Patients were required to be hemodynamically stable at the time of transfusion to be included in the analysis. Patients who received either a single-unit or double-unit transfusion for asymptomatic anemia at any time during the index admission were included. Hemodynamic stability was defined as patients without tachycardia (pulse < 100 bpm) and who were normotensive (systolic blood pressure >/= 90 mm Hg). Patients with ongoing vasopressor requirements were excluded. Additionally, patients who received perioperative transfusion for acute blood loss or active bleeding were excluded. Any patient who was hemodynamically unstable, pregnant, incarcerated, or under the age of 18 years old was excluded from the study. Thus, asymptomatic anemia was defined as hemoglobin levels between < 7 g/dL and ≥ 6 g/dL, hemodynamically stable, with no vasopressor requirements, no perioperative transfusion for acute blood loss, and no active bleeding.

Initial patient lists were retrieved from electronic medical records (EMR). Patient lists were retrieved from the local trauma registry during the previously mentioned dates. Demographic and clinical data were imported directly from the EMR into the Health Insurance Portability and Accountability Act (HIPAA)-compliant electronic data capture tool, REDCap (Vanderbilt University, Nashville, TN). Trained and monitored abstractors collected and entered data on the number of PRBC units transfused, operative interventions following transfusion, pre- and post-transfusion hemoglobin and hematocrit levels, and pre- and post-transfusion vital signs. In addition, if an additional transfusion(s) was needed following the index transfusion at any time during admission, this was recorded. Charts were reviewed by two independent abstractors, with any discrepancy in data being analyzed by a third abstractor.

Study data were managed using REDCap electronic data capture tools. All analyses were performed using R version 4.4.3 (R Foundation for Statistical Computing, Vienna, Austria). All categorical data are presented as counts with associated percentages, and proportions were compared with a chi-square statistic. Continuous variables are presented as the median with 25th and 75th interquartile range (IQR). Differences between groups for continuous variables were compared with a Wilcoxon rank-sum test. For all statistical tests, a two-sided p-value of < 0.05 was considered statistically significant. Clinically relevant variables with a univariable p-value <0.20 were entered into an initial multivariable logistic regression model to determine the impact of transfusion strategy on the need for subsequent transfusion.

## Results

Over the study period, 344 patients met the inclusion criteria as hemodynamically stable, non-bleeding, anemic trauma patients who received either a single-unit or double-unit PRBC transfusion. A total of 230 (67%) patients received a single-unit PRBC transfusion, and 114 (33%) patients received a double-unit PRBC transfusion. Observed agreement was 99% (342/344). Cohen’s k was 0.99, indicating almost perfect agreement. The discrepancies were resolved by consensus. There were no missing data.

Baseline characteristics and patient presentation group comparisons for the single-unit and double-unit groups are described in Table 1. These values represent data on admission, not the time of transfusion. The overall median age was 45 years, 246 (72%) of whom were male. The most common mechanisms of injury were motor vehicle collision (122, 35%), followed by firearm (95, 28%), pedestrian versus auto (43, 13%), and fall (40, 12%). Baseline characteristics were not statistically different between groups. The median injury severity score was 17 (11, 26) in the double-unit group and 20 (14, 27) in the single-unit group. Admission blood pressure, pulse, and Glasgow Coma Scale (GCS) score were not significantly different between groups, nor was the need for inotropic support or oxygen support on admission.

**Table 1:** Comparison of patient presentation with asymptomatic anemia treated with single- vs. double-unit PRBC transfusion. MVC, motor vehicle collision; Ped vs. Auto, pedestrian versus automobile; MCC, motorcycle collision; BP, blood pressure; GCS, Glasgow Coma Scale. * Pearson’s chi-squared test; # Wilcoxon rank-sum test; ## Fisher’s exact test used because of sparse cell counts (Fisher’s does not produce a conventional numeric test statistic; therefore, only the p-value is reported for variables analyzed using Fisher’s exact test). Odds ratios for mechanism of injury were estimated using logistic regression and are reported in the Appendices.

	Overall (N = 344)	Double unit (N = 114)	Single unit (N = 230)	Test statistic	p-value
Age, median (IQR)	45 (27, 63)	45 (28, 62)	45 (27, 63)	13051^#^	>0.9
Sex (male), n (%)	246 (72%)	84 (74%)	162 (70%)	0.25^*^	0.5
Blunt injury, n (%)	244 (71%)	77 (68%)	167 (73%)	0.72^*^	0.3
Mechanism of injury, n (%)				^##^	0.2
MVC	122 (35%)	34 (30%)	88 (38%)		
Firearm	95 (28%)	35 (31%)	60 (26%)		
Ped vs. Auto	43 (13%)	18 (16%)	25 (11%)		
Fall	40 (12%)	15 (13%)	25 (11%)		
MCC	21 (6.1%)	5 (4.4%)	16 (7.0%)		
Other	8 (2.3%)	1 (0.9%)	7 (3.0%)		
Other land transport	6 (1.7%)	1 (0.9%)	5 (2.2%)		
Cut pierce	5 (1.5%)	2 (1.8%)	3 (1.3%)		
Crush	4 (1.2%)	3 (2.6%)	1 (0.4%)		
Injury Severity Score, median (IQR)	19 (13, 27)	17 (11, 26)	20 (14, 27)	11839.5^#^	0.2
Admission systolic BP, median (IQR)	118 (93, 140)	118 (97, 140)	118 (92, 140)	13604^#^	0.5
Admission pulse, median (IQR)	104 (84, 122)	103 (84, 122)	105 (85, 122)	12653^#^	0.7
Admission GCS, median (IQR)	15 (10, 15)	15 (12, 15)	15 (9, 15)	14418.5^#^	0.10
Admission need for inotropic support, n (%)	47 (14%)	12 (11%)	35 (15%)	1.05^*^	0.2
Admission oxygen requirement, n (%)	244 (71%)	80 (70%)	164 (71%)	0.01^*^	0.8

Outcomes are described in Table 2. Median hemoglobin was significantly higher prior to transfusion in the single-unit group (6.70 g/dl; 6.40, 6.90) versus the double-unit group (6.30 g/dl; 5.80, 6.70). Likewise, the median hematocrit was significantly higher in the single-unit group (20.55%; 19.60, 21.50) versus the double-unit group (19.20%; 17.80, 20.50). Vital signs prior to transfusion were not significantly different between groups.

**Table 2:** Comparison of outcomes between those receiving single- vs. double-unit PRBC. Hgb, hemoglobin; Hct, hematocrit; PRBC, packed red blood cells; LOS, length of stay. * Pearson’s chi-squared test; # Wilcoxon rank-sum test; ## Fisher’s exact test used because of sparse cell counts (Fisher’s does not produce a conventional numeric test statistic; therefore, only the p-value is reported for variables analyzed using Fisher’s exact test). The odds ratio for transfusion reaction was estimated from the 2 × 2 Fisher’s exact test and is reported in the Appendices.

	Overall (N = 344)	Double unit (N = 114)	Single unit (N = 230)	Test statistic	p-value
Most recent Hgb prior to administration, median (IQR)	6.60 (6.20, 6.90)	6.30 (5.80, 6.70)	6.70 (6.40, 6.90)	7772^#^	<0.001
Most recent Hct prior to administration, median (IQR)	20.10 (19.00, 21.30)	19.20 (17.80, 20.50)	20.55 (19.60, 21.50)	7632.5^#^	<0.001
First Hgb post-administration, median (IQR)	8.00 (7.55, 8.65)	8.30 (7.70, 9.30)	7.90 (7.50, 8.40)	16804^#^	<0.001
Most recent pulse prior to administration, median (IQR)	96 (82, 108)	96 (81, 108)	96 (82, 109)	13193^#^	>0.9
Most recent systolic blood pressure prior to administration, median (IQR)	122 (109, 138)	120 (108, 139)	123 (109, 138)	12903.5^#^	0.8
Most recent O2 saturation prior to administration, median (IQR)	98 (96, 100)	99 (97, 100)	98 (96, 100)	13988^#^	0.3
First Hct post-administration, median (IQR)	24.45 (22.80, 26.50)	25.30 (23.10, 27.80)	24.20 (22.80, 25.90)	15693^#^	0.003
PRBC in the first 24 hours (# of units from 0 to 24 hours), median (IQR)	1 (0, 3)	1 (0, 3)	1 (0, 3)	12866.5^#^	0.8
PRBC in the first 72 hours (# of units total from 0 to 72 hours), median (IQR)	2 (1, 4)	3 (1, 4)	2 (1, 4)	14639.5^#^	0.074
Total # PRBC units, median (IQR)	3 (2, 7)	4 (2, 7)	3 (1, 6)	15340.5^#^	0.005
ICU LOS (days), median (IQR)	6 (3, 15)	5 (2, 11)	6 (3, 16)	11396.5^#^	0.048
Hospital LOS (days), median (IQR)	16 (10, 28)	14 (8, 28)	17 (10, 28)	12012^#^	0.2
30-day mortality, n (%)	38 (11%)	11 (9.6%)	27 (12%)	0.16^*^	0.6
Wound infection, n (%)	48 (14%)	12 (11%)	36 (16%)	1.27^*^	0.2
Transfusion reaction, n (%)	3 (0.9%)	1 (0.9%)	2 (0.9%)	^##^	>0.9
Post-transfusion operation required, n (%)	173 (50%)	62 (54%)	111 (48%)	1.04^*^	0.3
Additional transfusion required, n (%)	138 (40%)	48 (42%)	90 (39%)	0.17^*^	0.6

The absolute increase in hemoglobin was greater in those who received two units compared to those who received one unit of PRBC (2.1 vs. 1.3 g/dL, p < 0.001), and the double-unit transfusion group received more blood transfusions over the course of hospitalization than the single-unit group (4 vs. 3 units, p = 0.005). The need for additional transfusions did not differ between groups (48 (42%) vs. 90 (39%), p = 0.6).

Hospital LOS was not significantly different between groups, with a median of 17 (10, 28) in the single-unit group and 14 (8, 28) in the double-unit group. ICU LOS was six (3, 16) in the single-unit group and five (2, 11) in the double-unit group, which was statistically significant. The total number of PRBC units was significantly higher in the double-unit group (4; 2, 7) compared to the single-unit group (3; 1, 6). Mortality, wound infection, and transfusion reaction were not significantly different between groups.

Clinically relevant variables with a univariable p-value <0.20 were entered into an initial multivariable logistic regression model. Backward stepwise selection using the step() function in R was performed based on the Akaike Information Criterion (AIC). The single-unit versus double-unit transfusion strategy was retained in the final multivariable model regardless of statistical significance as the primary exposure. Backward stepwise selection was applied only to the remaining candidate covariates, and adjusted and unadjusted odds ratios for variables retained in the final model are presented in Table 3. The final model identified only post-transfusion operative intervention (i.e., surgical intervention performed after transfusion for anemia) as an independent predictor of repeat transfusion, with an adjusted odds ratio (95% CI) of 6.55 (3.99, 10.76; p < 0.001). No other independent predictors were identified. After adjusting for post-transfusion operation, most recent hemoglobin, and admission GCS score, the initial transfusion strategy did not significantly affect the need for repeat transfusion, with an adjusted odds ratio (95% CI) of 0.93 (0.54, 1.59; p = 0.786).

**Table 3:** Adjusted OR for additional PRBC transfusion. Hgb, hemoglobin; PRBC, packed red blood cell; GCS, Glasgow Coma Scale; OR, odds ratio; CI, confidence interval.

Variable	Unadjusted OR (95% CI)	Unadjusted p	Adjusted OR (95% CI)	Adjusted p
Post-transfusion operation	6.40 (3.93, 10.41)	<0.001	6.55 (3.99, 10.76)	<0.001
Most recent Hgb	0.96 (0.67, 1.36)	0.803	0.79 (0.52, 1.21)	0.279
Admission GCS	0.97 (0.93, 1.02)	0.224	0.98 (0.93, 1.04)	0.551
Double-unit transfusion	1.11 (0.70, 1.75)	0.662	0.93 (0.54, 1.59)	0.786

## Discussion

The primary findings in the current study suggest that a single-unit transfusion strategy has similar patient outcomes to a double-unit transfusion strategy for non-bleeding anemic trauma patients, which aligns with previous research. Hospital-wide initiatives aimed at reducing transfusion volume have shown consistent success through the implementation of single-unit transfusion protocols. At one major academic institution, a shift to a single-unit default policy led to a 13.9% reduction in PRBC utilization, with an additional 8.3% decrease following the adoption of a more restrictive hemoglobin threshold, all without negatively impacting patient outcomes or prolonging LOS [9]. Broad reviews of transfusion guidelines and clinical data support these findings; in an analysis of 26 guidelines and 14 clinical studies, single-unit strategies were associated with a 17-44% reduction in blood use without any increase in mortality or complications [10].

The current study found no differences in hospital LOS or 30-day mortality, and the median number of total PRBC units was significantly lower in the single-unit group (3; 1, 6) compared to the double-unit group (4; 2, 7). These findings align with prior research. In a previous systematic review, 92.3% of articles recommended a single-unit strategy, followed by rechecking the patient’s Hgb level and considering their clinical presentation before further transfusion, noting no statistically significant difference in length of hospital stay or 30-day mortality rate and decreased PRBC utilization by 10-41% with the use of a single-unit strategy [10]. Similar findings have been noted in patients with hematologic malignancies, with no significant difference between those receiving single- versus double-unit transfusions in categories such as LOS, 30-day mortality, readmissions, and in-hospital deaths [11]. Likewise, a randomized trial conducted with post-partum anemic mothers showed no difference in 30-day complications such as maternal LOS, ICU admissions, wound infections, or venous thromboembolism when comparing single versus multi-unit transfusions [12]. Additionally, both studies showed a statistically significant decrease in PRBC utilization after implementation of single-unit transfusion strategies [11,12].

The current study identified significantly lower total PRBC utilization during hospitalization in the single-unit group than in the double-unit group (median, 3 vs. 4 units). Assuming an estimated blood acquisition cost of approximately $550 per PRBC unit, the observed difference in median total PRBC utilization corresponds to an estimated direct blood product cost of approximately $1,650 for patients managed with an initial single-unit strategy compared with $2,200 for those managed with an initial double-unit strategy. Although a formal economic evaluation was beyond the scope of this study and actual patient-level costs were not assessed, these findings suggest that an initial single-unit transfusion strategy may reduce overall blood product utilization and the associated acquisition costs during hospitalization.

The regression analysis identified surgical intervention following transfusion for anemia as an independent predictor of repeat transfusion. Similar findings were seen in a recent meta-analysis, in which head and neck surgery patients with lower Hgb had a higher rate of transfusion compared to non-anemic patients [13]. Whether or not a double-unit transfusion may reduce repeat transfusions in anemic patients postoperatively remains to be determined. A consensus statement from the International Consensus Conference on Anemia Management in Surgical Patients (ICCAMS) recommended PRBC transfusion postoperatively for severe anemia when other options may not be clinically sufficient, noting that PRBC transfusion is an independent risk factor for morbidity and mortality and the combination of anemia and transfusion contributes to poor outcomes [14]. It is also worth noting that the current study found that the double-unit group had a significantly lower median ICU LOS, but had a p-value of 0.048 and was not corrected for multiplicity and therefore should be interpreted cautiously as an exploratory outcome. Thus, additional research may be warranted to determine the risk-to-benefit ratio for a double-unit transfusion in anemic trauma patients (Hgb < 7 g/dl) prior to planned surgical intervention.

To our knowledge, no other studies specifically examining single- versus double-unit PRBC transfusion in trauma patients with asymptomatic anemia have been published. In a recent systematic review, outcomes for single- versus double-unit in broader populations were null with low certainty of evidence [15]. The findings of the current study are consistent with these observations and suggest that, similar to non-trauma population studies, a single-unit transfusion strategy has similar outcomes to a double-unit transfusion strategy while also reducing costs and saving a limited resource.

The primary limitation of this study is that it is retrospective. This precludes the exclusion of selection bias and unevaluated differences as potential confounding variables. This allows only for associations to be made and cannot account for potential confounding differences. In addition, there were initial differences in hemoglobin prior to transfusion between the two groups, with a lower hemoglobin in the double-unit group. Thus, the transfusion strategy for sicker patients was more likely to be double-unit rather than single, though this was adjusted for in the multivariable regression. The decision to transfuse trauma patients with a hemoglobin concentration of < 7 g/dL, as well as the decision to administer an initial single- or double-unit PRBC transfusion, was made at the discretion of the treating clinician. Because the specific clinical rationale for transfusion and the selection of transfusion volume were not captured, residual confounding by indication cannot be excluded. Furthermore, baseline clinical variables were collected at admission rather than immediately before transfusion. Although this approach allowed comparison of patient characteristics at presentation, these measurements may not fully reflect patients’ physiologic status at the time transfusion decisions were made. However, all included patients were required to be hemodynamically stable without vasopressor requirements before transfusion, reducing variability in pre-transfusion hemodynamic status across the study population. Finally, since this study examined trauma patients exclusively from a single trauma center, application of the results to other populations should be done cautiously, especially when treatment modalities other than those described above are applied.

## Conclusions

A single-unit transfusion strategy in stable, non-bleeding trauma patients demonstrated safety and efficacy for achieving an adequate hematologic response. Furthermore, the single-unit transfusion strategy did not increase the likelihood for the need of additional subsequent transfusions. Patients initially treated with a double-unit strategy received a greater total number of PRBC units during hospitalization, which has been associated with increased risk to the patient and reduces a limited resource for trauma care. This study supports a single-unit transfusion strategy, specifically in trauma populations, and extends observations from broader patient populations.

## Appendices

**Table 4:** Odds ratios for mechanism of injury and transfusion reaction. MCC, motorcycle collision; Ped vs. Auto, pedestrian versus automobile. Fisher’s exact test was used for categorical comparisons with sparse cell counts and does not produce a conventional test statistic. Odds ratios for mechanism of injury were estimated separately using logistic regression, with motor vehicle collision (MVC) as the reference category. For transfusion reaction, the odds ratio and 95% confidence interval were estimated from the 2 × 2 Fisher’s exact test.

	Odds ratio	95% Confidence interval	p-value
MCC	0.81	0.25, 2.25	0.70
Fall	1.55	0.72, 3.28	0.25
Firearm	1.51	0.85, 2.69	0.16
Ped vs. Auto	1.86	0.90, 3.84	0.92
Other land transport	0.52	0.03, 3.37	0.55
Crush	7.76	0.96, 159.96	0.08
Cut pierce	1.72	0.22, 10.85	0.56
Other	0.37	0.02, 2.19	0.36
Transfusion reaction	1.01	0.02, 19.58	>0.9
